# Analysis of the Long Non-Coding and Messenger RNA Expression Profiles in the Skin Tissue of Super Merino and Small-Tailed Han Sheep

**DOI:** 10.3390/cimb46090570

**Published:** 2024-08-31

**Authors:** Jiaqi Fu, Xinyu Zhang, Dan Wang, Wenqing Liu, Caihong Zhang, Wei Wang, Wei Fan, Lichun Zhang, Fuliang Sun

**Affiliations:** 1 College of Agriculture, Yanbian University, Yanji 133000, China; 2022010659@ybu.edu.cn (J.F.); 2021010666@ybu.edu.cn (X.Z.); 2021010661@ybu.edu.cn (D.W.); 2023010683@ybu.edu.cn (W.L.); 2023010692@ybu.edu.cn (C.Z.); 2023050989@ybu.edu.cn (W.W.); 2023050972@ybu.edu.cn (W.F.); 2 Institute of Animal Biotechnology, Jilin Academy of Agricultural Sciences, Gongzhuling 136100, China; zhang_lich@163.com

**Keywords:** super merino sheep, small-tailed han sheep, lncRNA, mRNA, RNA sequencing, immunohistochemistry

## Abstract

Wool quality and yield are two important economic livestock traits. However, there are relatively few molecular studies on lncRNA for improving sheep wool, so these require further exploration. In this study, we examined skin tissue from the upper scapula of Super Merino (SM) and Small-Tailed Han (STH) sheep during the growing period. The apparent difference was verified via histological examination. High-throughput RNA sequencing identified differentially expressed (DE) long non-coding (lncRNAs) and messenger RNAs (mRNAs). The target gene of DE lncRNA and DE genes were enrichment analyzed using Gene Ontology (GO) and the Kyoto Encyclopedia of Genes and Genomes (KEGG). A Reverse Transcription quantitative Polymerase Chain Reaction (RT-qPCR) was used to verify randomly selected DE lncRNAs and mRNAs. Finally, the DE, *RAC2*, *WNT11*, and *FZD2* genes, which were enriched in the Wnt signaling pathway, were detected via immunohistochemistry. The results showed that a total of 20,888 lncRNAs and 31,579 mRNAs were identified in the skin tissues of the two sheep species. Among these, 56 lncRNAs and 616 mRNAs were differentially expressed. Through qRT-PCR, the trends in the randomly selected DE genes’ expression were confirmed to be aligned with the RNA-seq results. GO and KEGG enrichment analysis showed that DE lncRNA target genes were enriched in GO terms as represented by epidermal and skin development and keratin filature and in KEGG terms as represented by PI3K-Akt, Ras, MAPK, and Wnt signaling pathways, which were related to hair follicle growth and development. Finally, immunohistochemistry staining results indicated that *RAC2*, *WNT11*, and *FZD2* were expressed in dermal papilla (DP). The lncRNAs *MSTRG.9225.1* and *MSTRG.98769.1* may indirectly participate in the regulation of hair follicle growth, development, and fiber traits by regulating their respective target genes, *LOC114113396*(*KRTAP15-1*), *FGF1*, and *IGF1*. In addition, *MSTRG.84658.1* may regulate the Wnt signaling pathway involved in the development of sheep hair follicles by targeting *RAC2*. This study provides a theoretical reference for improving sheep breeding in the future and lays a foundation for further research on the effects of *MSTRG.84658.1* and the target gene *RAC2* on dermal papilla cells (DPC).

## 1. Introduction

Wool is an important and widely used raw material in the textile industry [1]. The Small-Tailed Han (STH) sheep, known for its fast growth and high breeding rates, is the dominant breed in northern China. However, for decades, there has been little work in improving the wool’s characteristics. Xinji sheep are a breed of fine-wool sheep first bred by the Jilin Academy of Agricultural Sciences and the Xinjiang Academy of Animal Husbandry Sciences in the 1980s. They were renamed Super Merino (SM) sheep in 2014 [2]. Both Super Merino and Small-Tailed Han sheep provide high-value wool, but SM have a higher wool quality, including a finer diameter, higher roll curvature, longer staple fiber length and greater yield. These traits are closely related to hair follicle growth. As a crucial component of the skin, hair follicles (HFs) dictate the wool’s yield and quality [3]; hair follicles are important organs controlling the growth and maturation of mammalian hair that can be divided into primary and secondary hair follicles according to their structural characteristics and developmental stages [4]. Hair follicles’ morphological growth is a multifaceted process entailing an interplay between epithelial cells and dermal fibroblasts [5]. The initial signal from the mesenchymal cells prompts the epidermis to develop into hair embryos, which release specific elements that trigger the development of dermal fibroblasts and mastoids. Then, the dermal papillae release the “second signal”, encouraging the growth and diversification of epithelial cells; this forms a well-structured hair follicle [6].

Long non-coding RNAs (lncRNAs) refer to a category of RNA strands exceeding 200 nucleotides in length and incapable of encoding proteins [7]; in some contexts, they may be more specific than protein-coding genes [8], and they have been confirmed to participate in biological activities, including cell proliferation and differentiation and programmed cell death [9]. lncRNAs can be classified into antisense, intron, intergene, and sense lncRNAs, according to their positional relationship with protein-coding genes [10]. Research has indicated that lncRNAs have a significant function in controlling gene expression within organisms, but they are rarely annotated in the sheep genome [11]. Many functional genes and non-coding RNAs are involved in the regulation of fiber yield and quality in cashmere [12,13]. Therefore, wool characteristics can feasibly be enhanced by regulating lncRNA expression [14].

Recently, with the emergence of molecular breeding techniques and the discovery of lncRNA functions, increasing numbers of researchers have begun focusing on the molecular mechanisms determining fiber traits in economically valuable animals used for their wool. One study showed that genes such as *KRT26*, *KRT28*, and lncRNAs (such as *MSTRG.16794.17* and *MSTRG.17532.2*) have potentially important roles in regulating diameter in cashmere [15]. Jin et al. found that, through controlling *ITGB5*, *TlN2*, *CTSS*, and additional proteins that play a role in cell proliferation, lncRNA *MTC* promoted the production of Liaoning wool goat skin fibroblasts [16]. Sun et al. found that the lncRNAs *MSTRG.12818.1* and *MSTRG.13824.1* play significant roles in regulating the hair follicle growth stage in Doper sheep, while lncRNA *MSTRG.15931.1* aids in regulating the termination phase [17]. Yin et al. found that *lncRNA-599554* contributes to dermal papilla cell induction in cashmere goats, which may be achieved by promoting *Wnt3a* expression via *miR-15b-5p* sponging [18]. In the current database, most of the identified lncRNAs come from humans and mice [19]; as such, those of other species still require further exploration.

Although there has been some progress in lncRNAs in terms of regulating hair phenotype and follicle growth and development, they still need to be further explored and studied. In this study, high-throughput sequencing was performed on skin tissues from Super Merino and Small-Tailed Han sheep in order to screen for differentially expressed lncRNAs and mRNAs. In addition, they were systematically analyzed to explore their potential roles. This study provides a theoretical basis for further exploring the molecular mechanism of lncRNAs that regulate hair follicle growth and development.

## 2. Materials and Methods

### 2.1. Sample Collection and Ethical Statement

In June, six 6-month-old Super Merino (SM) and six Small-Tailed Han sheep (STH) were selected from the Animal Husbandry Branch of the Jilin Academy of Agricultural Sciences. To reduce the environmental impact, both groups of sheep were raised in the same environment, using the same feed and hay formulas. All skin tissue samples were collected in mid-October of the same year, when the wool cycle was expected to be in its growing phase, and two approximately 1 cm^2^ pieces of skin tissue were collected from the upper shoulder via surgical operation; one of these pieces was placed in paraformaldehyde for fixing, and the other piece was quickly frozen in liquid nitrogen and stored at −80 °C. The metal surgical instruments used were high-temperature sterilized and treated with 0.1% DEPC water. This research scheme was approved by the Experimental Animal Welfare Ethics Committee of Yanbian University (YD20220608002, 8 June 2022).

### 2.2. Histomorphological Study

The collected skin samples were placed in 4% paraformaldehyde for 24 h. The samples were rinsed with running water for 12–24 h. Then, the samples were subjected to 75%, 85%, 90%, 95%, and 100% alcohol gradients for dehydration. Anhydrous ethanol was mixed with xylene in equal amounts for 1 h and then placed in xylene for 20 min for transparency treatment. The tissue samples were then immersed in soft wax for 1 h, before being immersed in hard wax for 1.5 h; finally, they were embedded both laterally and longitudinally. The paraffin blocks were then sliced after drying, stained with hematoxylin-eosin and then observed and preserved under an Olympus BX53 biological microscope (OLYMPUS, Tokyo, Japan). We used ImageJ software (Version 1.8.0) to measure hair follicle traits, with 6 biological and 3 technical replicates for each measurement.

### 2.3. RNA Isolation, Library Preparation, and Sequencing

Sheep skin tissue samples were removed from the −80 °C refrigerator, placed in liquid nitrogen and then ground in a mortar. Trizol reagent (Sangon Biotech, Shanghai, China) was used to extract the total RNA from the skin tissue, and the concentration of the extracted nucleic acid was detected with a Nanodrop2000 (Thermo Fisher Scientific, Waltham, MA, USA). The sample integrity was tested using an Agilent 2100 (Agilent Technologies, Santa Clara, CA, USA). The VAHTSTM Small RNA Library Prep Kit for Illumina (Illumina, San Diego, CA USA) was used to construct the library, and the Qsep-400 method was used for quality inspection. Mapped data were acquired using Sequencing-By-Synthesis (SBS) technology, employing the Illumina HiSeq high-throughput sequencing system for cDNA library sequencing and aligning fresh data with the designated reference genome (Ovis_aries.GCF_016772045.1_ARS_UI_Ramb_v2.0.genome.fa).

### 2.4. lncRNA Identification and Target Gene Prediction

The prediction of new lncRNAs consisted of two parts: basic and potential coding ability screening. Basic screening involved selecting transcripts whose class_code was “i”, “x”, “u”, “o”, or “e” [20]. Transcripts with a length ≥ 200 bp and ≥2 exons were selected. Three parts of the transcript with fragments per kilobase of transcript per million fragments mapped (FPKM) ≥ 0.1 were selected [21]. Because lncRNAs do not encode proteins, their transcripts must be determined through screening and confirming their coding potential. In order to forecast target genes, it is necessary to rely on the operational mechanisms of lncRNAs and the genes, employing two distinct prediction techniques. The first prediction method assumes that neighboring genes within 100 kb of the lncRNA are its cis-target genes. The alternative approach entails forecasting the lncRNA’s trans-target genes through examining the relationship between the lncRNA and mRNA expression levels of the samples.

### 2.5. Expression Analysis

edgeR [22] is used for analyzing differential expression across different samples (groups) and is applicable for such analyses regardless of biological duplication. StringTie (1.3.1) [23] was used to calculate the FPKMs of both lncRNAs and the coding genes in each sample. Gene FPKMs were computed by summing the FPKMs of the transcripts in each gene group. FPKM is defined as the fragments per kilobase of exon per million fragments mapped, calculated based on the length of the fragments and read counts mapped to the fragment. In the process of detecting differentially expressed lncRNAs and mRNAs, a fold change ≥2 and False Discovery Rate (FDR < 0.01) were used as screening criteria. The fold change refers to the expression ratio between two samples (groups). In this study, genes with a high expression in SM were up-regulated genes, while genes with a high expression in STH were down-regulated genes.

### 2.6. Enrichment Analyses with Gene Ontology (GO) and Kyoto Encyclopedia of Genes and Genomes (KEGG) 

ClusterProfiler [24] was used to conduct an enrichment analysis of the biological processes, molecular functions, and cell components of the cis–trans-target genes and mRNAs that were differentially expressed among the samples.

The KEGG database helps researchers to input genes and express information in a whole network. As the main public database on pathways (Kanehisa, 2008 [25]), it not only provides all possible metabolic pathways, but also an extensive list of the enzymes responsible for catalyzing each reaction phase. Consequently, it serves as an effective instrument for analyzing metabolism and studying metabolic networks in living organisms.

### 2.7. Construction of PPI and lncRNA–mRNA Network

The protein–protein interaction (PPI) network of DE mRNAs was constructed using STRING and then visualized using Cytoscape software(Version 3.10.0) [26]. Due to their prediction mode, there are two types of lncRNA target genes: cis and trans. Based on the prediction and differential expression analysis results for target genes, we used Cytoscape to construct the regulatory networks of lncRNA cis- and lncRNA trans-target genes.

### 2.8. qRT-PCR Verification

DE lncRNAs and mRNAs were randomly selected, and GAPDH was selected as the reference gene [27]. The Total RNA Extractor (Trizol, Shanghai, China) was used to extract the total RNA from the samples. The LnRcute LncRNA First-Strand cDNA Kit (Beijing, China) was used to reverse-transcribe the total RNA into cDNA for real-time quantitative Polymerase Chain Reaction (qRT-PCR). Then, the LnRcute LncRNA qPCR Kit was used for RT-qPCR. A 20 µL system was selected, which included 12 µL of PreMix and 0.5 µL each of forward and reverse primers. Then, 4 µL of cDNA and 3 µL of RNase-free ddH2O were used; the reaction procedures were set at 95 °C for a 1-time 3 min cycle, at 95 °C for 5 s, 60 °C for 10 s, and 72 °C for a 15 s cycle run 40 times. Melt curve analysis was performed at the end of the PCR cycle to determine the specificity of the intended primers. The primer sequences are shown in Table 1.

### 2.9. Immunohistochemistry Study

The sheep skin tissue samples were treated with immunohistochemistry. The tissue sections were dewaxed in xylene 3 times (15 min each time), dehydrated with gradient alcohol (100%, 85%, and 75% for 5 min each) and then washed with distilled water. After washing, the tissue sections were placed in EDTA antigen repair buffer (PH8.0) for antigen repair and then were placed in a microwave (P70D20TL-P4, Galanz, Foshan, China) oven for 8 min at medium heat, 8 min at no heat, and 7 min at medium-low heat. This process should prevent the excessive evaporation of the buffer. In order to block endogenous peroxidase, the slices were placed in a 3% hydrogen peroxide solution and incubated at room temperature away from light for 30 min. The slides were placed in PBS (PH7.4) and washed three times on a decolorizing shaking table (TSY-B, Servicebio, Wuhan, China) for 5 min each time. The tissue was uniformly covered with 3% bovine serum albumin (BSA) and covered at room temperature for 30 min. We added Rabbit anti-RAC2 Polyclonal Antibody (abs118784, Polyclonal, Absin, Shanghai, China), Frizzled 2 Rabbit pAb (XG-866230, Polyclonal, Shanghai Sig Biotechnology Co., Ltd., Shanghai, China), and Rabbit anti-Wnt11 (bs—8568—r, polyclonal antibody, Bioss, Beijing, China) with PBS diluted (1:100) onto the slide and stored flat in a wet box at 4 °C overnight. On the second day, the slide was placed in PBS (PH7.4) and washed 3 times using a decolorizing shaker for 5 min each time. Then, we added Goat Anti-Rabbit IgG H&L/HRP antibody (bs-0295-g-HRP, Polyclonal, Bioss, Beijing, China) and incubated for 60 min at room temperature. The slide was again placed in PBS (PH7.4) and washed using a decolorizing shaker 3 times for 5 min each time. Freshly prepared DAB color-developing liquid drops were added to the slide; the color developing time was controlled under the microscope, and then the slide was washed with water to terminate color development. Hematoxylin (G1004, Servicebio, Wuhan, China) was redyed for about 1 min, followed by a water rinse, hematoxylin differentiation solution (G1309, Servicebio, Wuhan, China) for 10 SEC, a water rinse, hematoxylin blue return solution (G1340, Servicebio, Wuhan, China) for 8 min, a water rinse, and, finally, the film was dehydrated and sealed. Light images of the stained tissue were obtained using a microscope (E100, Nikon, Japan). The sample images were analyzed using the Nikon DS-U3 imaging system (Nikon, Tokyo, Japan).

### 2.10. Data Analysis

SPSS26.0 statistical software was used to analyze the differences among the experimental groups, and a *t*-test was used to establish the statistically significant differences between the groups. The results are expressed as mean ± standard error, and *p* < 0.05 indicates significant differences. *p* < 0.01 indicates a very significant difference.

## 3. Results

### 3.1. Characterization of Sheep Wool and Follicle Traits

**Figure 1:**
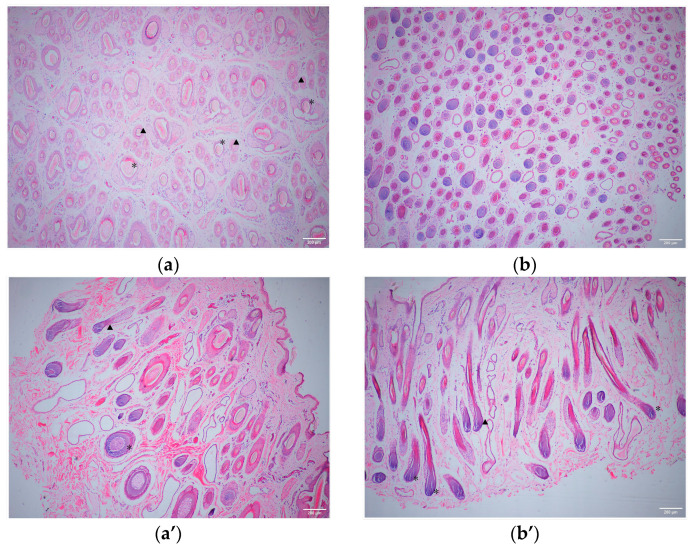
A 40× magnification histological analysis of the skin tissues of Small-Tailed Han sheep and Super Merino sheep. (**a**): Cross-section of the STH tissues. (**a’**): Transverse section of the STH tissues. (**b**): Cross-section of the SM tissues. (**b’**): Transverse section of the SM tissues. “★”: Primary hair follicle. “▲”: Secondary hair follicle.

The skin thickness, hair follicle density, and other data measured using ImageJ were analyzed using SPSS software (Version 26.0) (Table 2); the results showed that skin thickness and the primary and secondary hair follicle papilla diameters of the Small-Tailed Han sheep were significantly higher than those of the Super Merino sheep (*p* < 0.01), while the hair follicle density and S/P value of the Super Merino sheep were significantly higher than those of the Small-Tailed Han sheep (*p* < 0.01). The above statistical results were verified macroscopically using skin tissue sections (Figure 1).

### 3.2. Sequencing Data Quality Control

The sequencing of the 12 samples was completed, and 221 Gb of clean long non-coding RNA data were analyzed. The clean data per sample reached 17.07 Gb, and the percentage of Q30 bases was 93.62% or higher. A sequence alignment between the clean reads of each sample and the specified reference genome was performed, with efficiency ranging from 94.05% to 96.58% (Table 3). The findings showed the sequencing data to be of superior quality and suitable for further analysis.

### 3.3. Identification and Expression Analysis of lncRNA and mRNA

**Figure 2:**
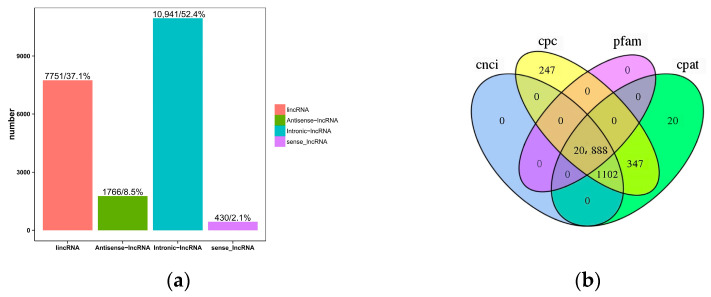
(**a**) Statistical map for predicting long non-coding RNAs. The horizontal coordinate indicates four different types of lncRNAs. The ordinate is the number of each lncRNA and its percentage in the total. (**b**) Prediction method: Venn diagram. Each circle represents a method for predicting lncRNA, and the number in the circle represents the number of transcripts predicted to be positive. The intersection of the four circles is taken as the prediction result.

The classification results for the four lncRNAs, predicted via CPC, CNCI, CPAT, and Pfam, are shown in Figure 2a. In order to display the analysis results intuitively, the non-coding transcripts, which were authenticated using the above four analysis software packages, were statistically analyzed, and a Venn diagram was drawn (Figure 2b). At the same time, combining the above four analysis results, a total of 20,888 lncRNAs were identified for subsequent analysis. In addition, 31,579 mRNAs were identified, of which 10,323 were novel genes. Among the 20,888 lncRNAs, 56 were differentially expressed, of which 15 were up-regulated, and 41 were down-regulated. Among the 31,579 mRNAs, 616 were differentially expressed, 210 were up-regulated, and 406 were down-regulated.

### 3.4. Comparative Analysis of lncRNA and mRNA Characteristics

**Figure 3:**
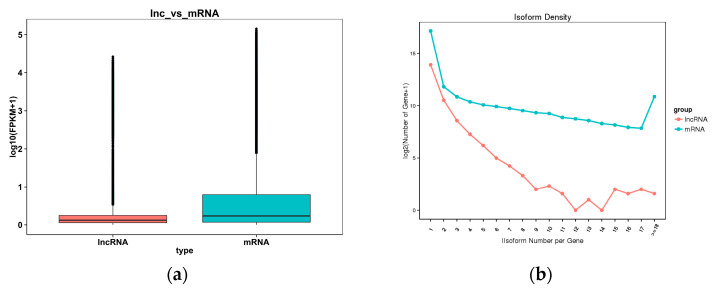
(**a**) A comparison of the lncRNA and mRNA expression levels. The horizontal coordinate is the molecular type, and the vertical coordinate is log10 (FPKM + 1). The box chart statistics are the maximum, upper quartile, median, lower quartile, and minimum, respectively. (**b**) A comparison of the lncRNA and mRNA variable shear isomers. The horizontal coordinate is the distribution of the number of variable shear isomers per gene, and the vertical coordinate is log2 (number of gene).

**Figure 4:**
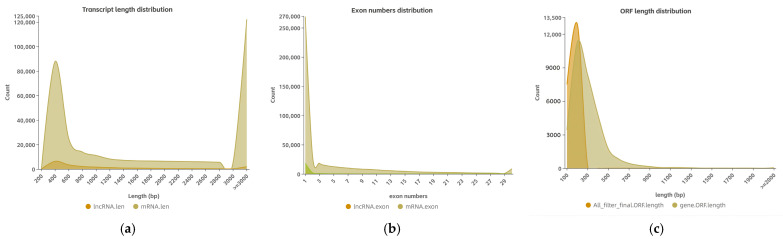
(**a**) Statistical diagram of the length distribution of mRNA and lncRNA. The horizontal coordinate is the length, and the vertical coordinate is the number of mRNAs or lncRNAs whose length is distributed within this range; the length unit is bp. (**b**) Statistical diagram of the number of exons corresponding to mRNAs and lncRNAs. The horizontal coordinate is the number of exons, and the vertical coordinate is the number of mRNAs or lncRNAs with exons distributed within this range. (**c**) Statistical map of ORF length corresponding to mRNAs and lncRNAs. The horizontal coordinate is the length, and the vertical coordinate is the mRNAs or the number of lncRNAs in this range.

A box diagram comparing the expression levels of the lncRNAs and mRNAs (Figure 3a) revealed that the expression level of the latter was higher than the former. The comparison results regarding the number of variable shear isomers showed that the number of mRNAs with the same number of variable cut isomers was higher than the number of lncRNAs (Figure 3b). An analysis comparing the length, exon count, and open reading frame (ORF) length between the lncRNAs and mRNAs (Figure 4a–c) showed that the former had a shorter sequence length, fewer exons, and a shorter ORF length compared with the latter.

### 3.5. PPI Network and Functional Analyses of DE mRNAs

**Figure 5:**
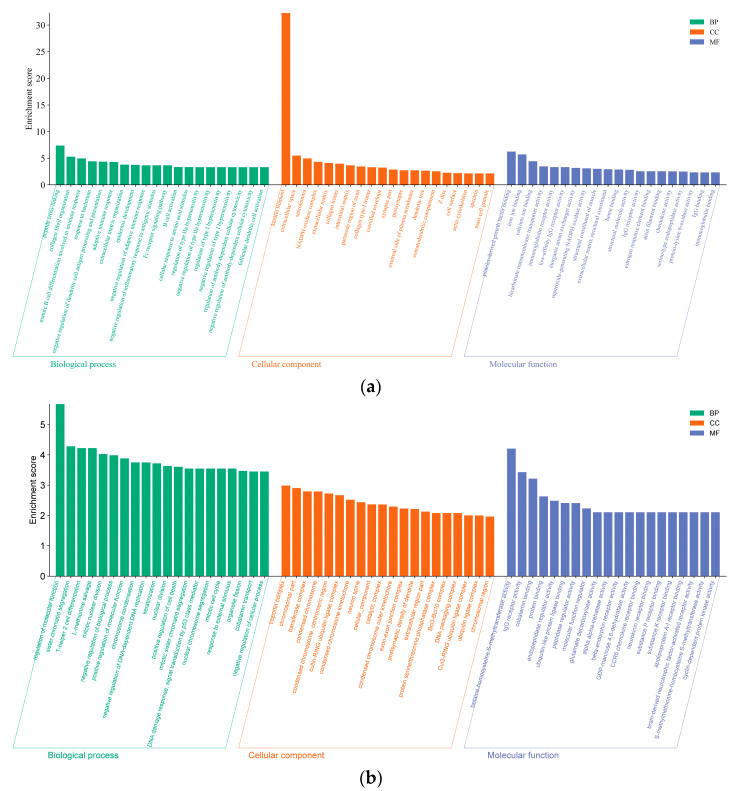

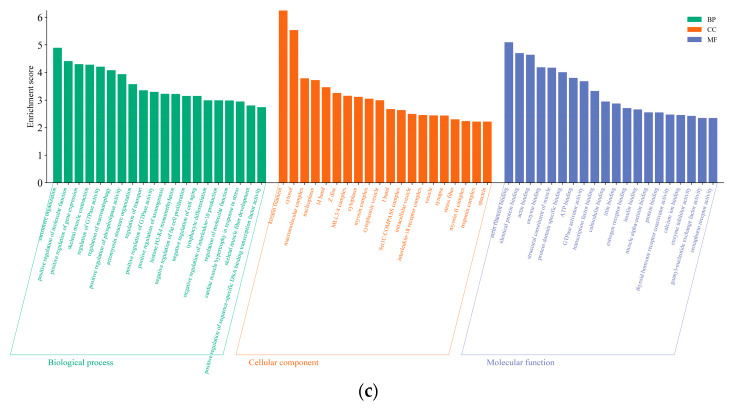
The top 20 enriched biological processes, cellular components, and molecular functions for differentially expressed lncRNA targets and mRNAs are listed. (**a**) Differentially expressed gene enrichment bar chart. (**b**) Differentially expressed lncRNA cis-target gene enrichment bar chart. (**c**) Differentially expressed lncRNA trans-target gene enrichment bar chart.

**Figure 6:**
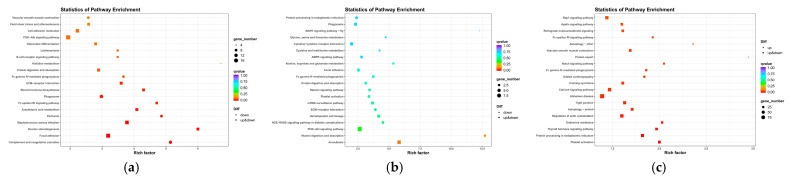
(**a**) The rich distribution diagram showing the differential expression of the mRNA KEGG channel. (**b**) The rich distribution diagram of the KEGG pathway of the lncRNA cis-target gene. (**c**) The rich distribution diagram showing the lncRNA trans-target gene KEGG pathway. The ordinate represents the name of the pathway, and the enrichment factor is the ratio of the proportion of differential genes that are annotated on a pathway to the proportion of all genes that are annotated to that pathway. The larger the enrichment factor, the more significant the enrichment level of differentially expressed genes or differentially expressed lncRNA cis–trans-target genes in this pathway. The color of the circle represents the qvalue, and the qvalue is the Pvalue after multiple hypothesis testing corrections. The smaller the qvalue, the more reliable the enrichment significance of differentially expressed genes in this pathway. The size of the circle indicates the number of genes enriched in the pathway, and the larger the circle, the more genes.

**Figure 7:**
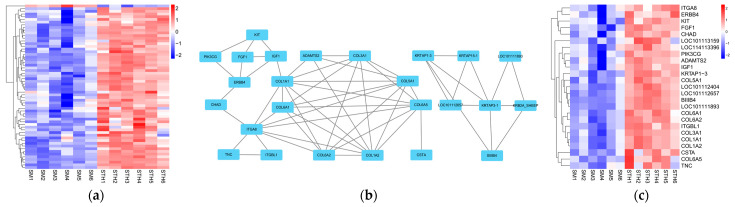
PPI network of DE mRNAs associated with hair follicle growth and development. (**a**) Clustering heatmap of 61 potential DE mRNA expression. (**b**) Constructed PPI network based on the interaction scores between DE mRNAs. Nodes represent proteins and edges represent a cluster heatmap of DE mRNA expression in PPI networks interacting with each other. (**c**) DE mRNA expression heatmap in PPI network.

GO annotation and KEGG enrichment were performed on 616 DE mRNAs to analyze potential functions and signaling pathways. The results showed that DE mRNAs were enriched in 2341 GO terms and 245 pathways. The top 20 significantly enriched terms and pathways are shown in Figure 5a and Figure 6a. The GO terms include three main processes: the biological process, cellular component, and molecular function. In the biological process, the *KRTAP15-1* was significantly enriched in terms of epidermis and skin development, keratinocyte differentiation, etc. (*p* < 0.05). In addition, the KEGG pathway enrichment results showed that the *FGF1* and *IGF-1* were significantly enriched in the ECM–receptor interaction and PI3K-Akt signaling pathway, two signaling pathways related to hair follicle growth and development (*p* < 0.05). Based on the above enrichment analysis, a total of 61 potential DE mRNAs (Figure 7a) related to hair follicle growth and development were selected, and PPI networks (Figure 7b) were then constructed. The DE mRNA expression profile that appears on the PPI network is shown as a heatmap (Figure 7c). *KRTAP15-1* is enriched in GO terms related to hair follicle growth and development, and it also interacts with *KRTAP1-3* and other differentially expressed genes. Similarly, *FGF1* and *IGF1* are enriched in pathways related to hair follicle growth and development, interacting with differentially expressed genes such as *KIT*. Therefore, it is speculated that *KRTAP15-1*, *FGF1*, *IGF-1*, and other DE mRNAs may play important roles in regulating the growth and development of sheep hair follicles.

### 3.6. GO and KEGG Analyses of DE lncRNA Target Genes

**Figure 8:**
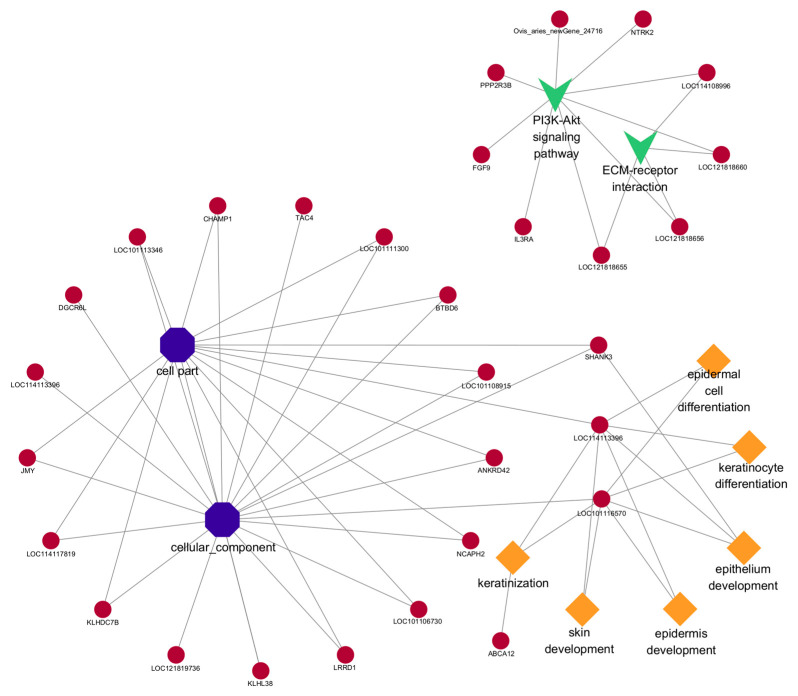
The network of cis-target genes of DE lncRNAs are enriched in important GO terms and pathways that may be related to hair follicle growth and development. The diamond represents biological processes; the octagon represents cellular components; “v” stands for pathway. The circle represents the cis-target gene.

**Figure 9:**
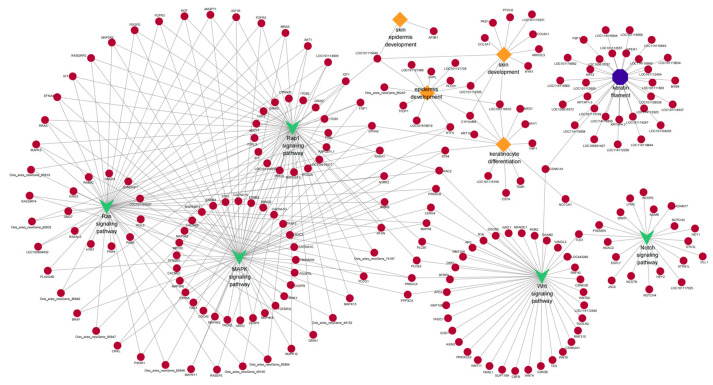
The trans-target genes of DE lncRNAs are enriched in important GO terms and pathways that may be related to hair follicle growth and development. The diamond represents biological processes; the octagon represents cellular components; “v” stands for pathway. The circle represents the cis-target gene.

Since there are two ways to predict lncRNA target genes, we conducted GO annotation and KEGG enrichment analysis on cis- and trans-target genes, respectively. Through analyzing the results for cis-target genes, GO terms and pathways related to hair follicle growth and development were discovered (*p* < 0.05) (Figure 8). The top 20 significantly enriched terms and pathways are shown in Figure 5b and Figure 6b. In the analysis of trans-target genes, GO terms and pathways related to hair follicle growth and development were also found (Figure 9). The top 20 significantly enriched terms and pathways are shown in Figure 5c and Figure 6c. The results showed that the enrichment results of differentially expressed lncRNA cis-target genes overlapped with those of differentially expressed mRNAs. For example, *KRTAP15-1* enriched epidermis and skin development, keratinocyte differentiation, and other GO terms according to both sets of analytical results. An enrichment analysis of trans-target genes showed that *FGF1*, *IGF1*, and *RAC2* were enriched in the Rap1, Ras, and MAPK signaling pathways related to hair follicle growth and development; *RAC2* was also enriched in the WNT signaling pathway. These results further suggest that *KRTAP15-1*, *FGF1*, *IGF1*, and *RAC2* may play important roles in hair follicle growth and development.

### 3.7. Regulatory Network

**Figure 10:**
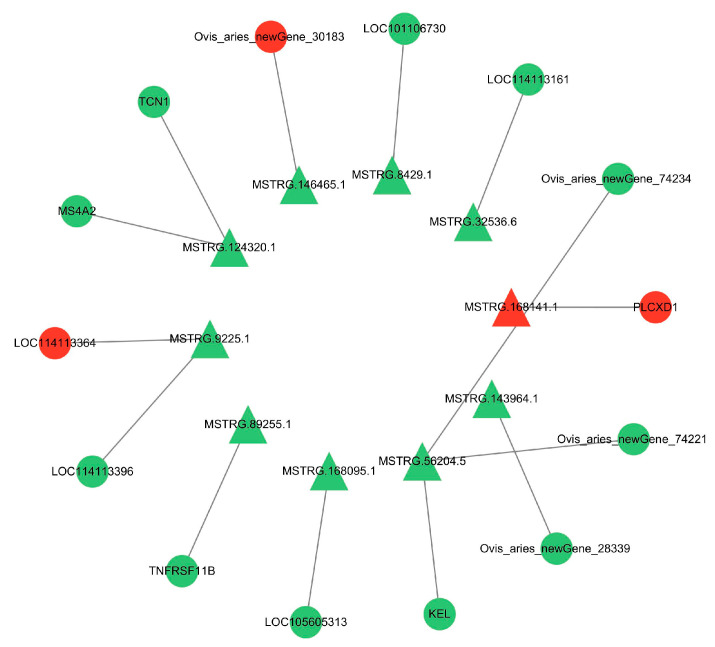
Interaction network diagram of the differentially expressed lncRNA and cis-target genes. Triangles are the DE lncRNAs. Circles are the DE mRNAs. Red is used for genes that are highly expressed in SM, and green is used for the same in STH.

**Figure 11:**
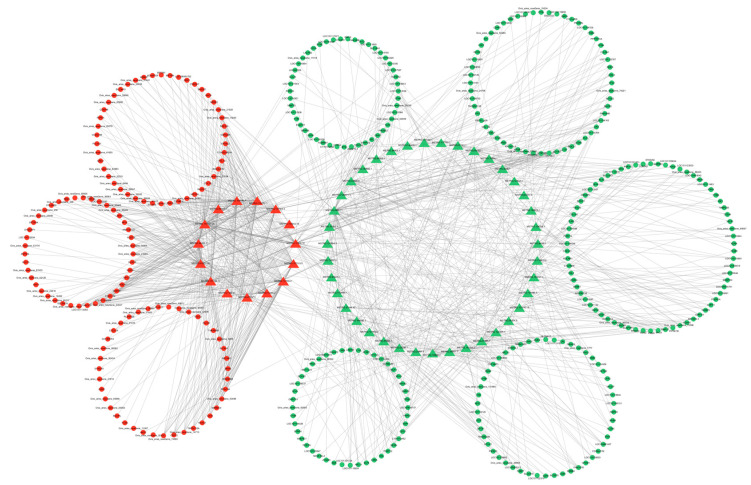
Interaction network diagram of the DE lncRNA and DE trans-target genes. Triangles are the differentially expressed lncRNAs. Circles are the DE mRNAs. Genes highly expressed in SM are denoted in red, while the same in STH are indicated in green.

Since there are two forms of lncRNA target genes, combining the predicted data of target genes with the differential expression data, we found that 10 DE lncRNAs had DE cis-target genes (Figure 10) and 54 DE lncRNAs had DE trans-target genes (Figure 11). The cis- and trans-target genes regulated by lncRNAs were visualized using Cytoscape software(Version 3.10.0). The results showed that *MSTRG.9225.1* had a cis-regulatory relationship with *KRTAP15-1*, *MSTRG.98769.1* had a trans-regulatory relationship with *FGF1*, and *IGF1* and *MSTRG.84658.1* had a trans-regulatory relationship with *RAC2*. These results suggest that the lncRNAs *MSTRG.9225.1*, *MSTRG.98769.1*, and *MSTRG.84658.1* may have a cis- or trans-regulatory relationship with their respective target genes.

### 3.8. Validation of the RNA-Seq Data

**Figure 12:**
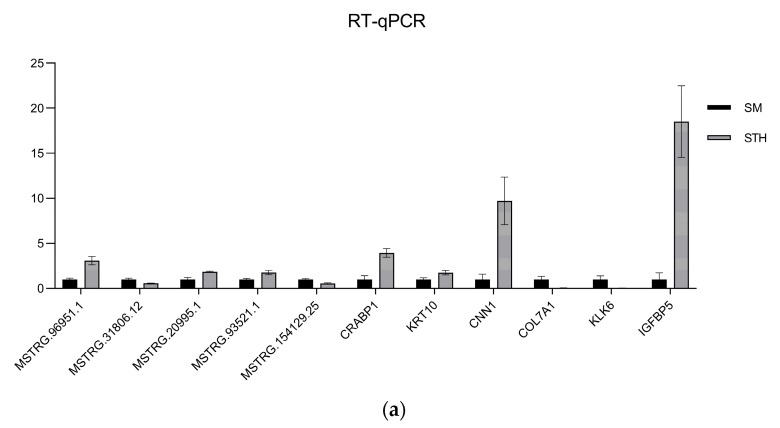

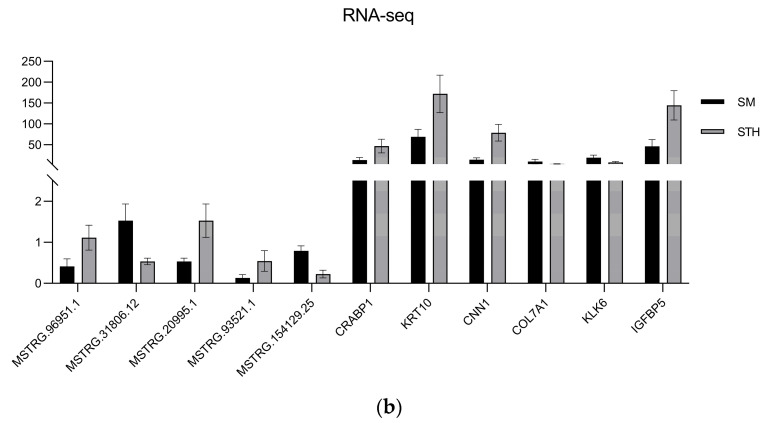
The results of qRT-PCR were compared with those of RNA-seq. (**a**) Histogram of lncRNA and mRNA’s qRT-PCR validation results. (**b**) Histogram of lncRNA and mRNA’s RNA-seq results. The horizontal coordinate indicates the gene ID, and the vertical coordinate indicates the relative expression of the gene.

To confirm the dependability of the RNA-seq findings, DE lncRNAs and mRNAs were randomly selected and their expression levels confirmed using qRT-PCR; finally, the qRT-PCR results were visualized using GraphPad Prism 9.5.1 software (Figure 12). A summary of the graph data is presented in Table 4. The outcomes demonstrated that the patterns observed in the DE lncRNA and DE mRNA expression patterns aligned with the RNA-seq outcomes, affirming the dependability of the RNA-seq data.

### 3.9. Immunohistochemistry Data

**Figure 13:**
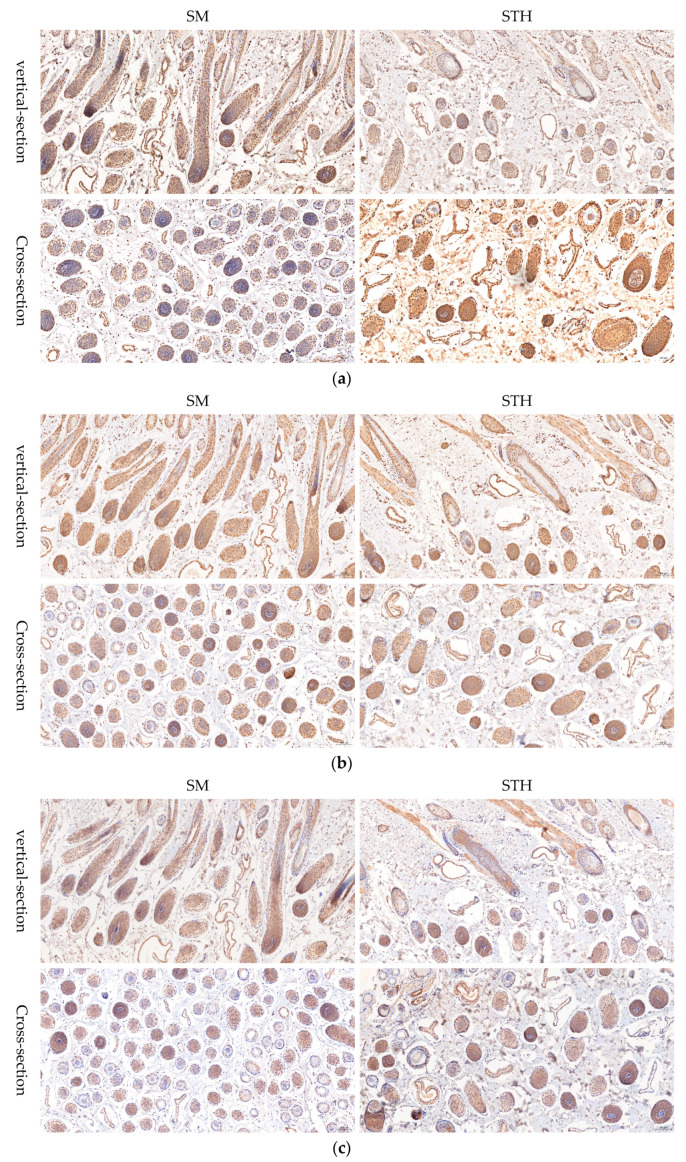
Light image of immunohistochemical analysis of skin tissue. (**a**) Rabbit anti-RAC2 Polyclonal Antibody. (**b**) Rabbit anti-Wnt11. (**c**) Frizzled 2 Rabbit pAb. The nucleus of the hematoxylin stain is blue, and the positive expression of DAB is brownish yellow.

In order to further study the differentially expressed genes enriched in the Wnt signaling pathway, immunohistochemical staining was used to detect the localization of *RAC2*, *WNT11*, and *FZD2* in skin tissue. The results showed that the differentially expressed genes *RAC2*, *WNT11*, and *FZD2* were expressed in the dermal papillae (DP) and outer root sheath (ORS) of both sheep species (Figure 13). This further suggests that *RAC2*, *WNT11*, and *FZD2* may play important regulatory roles in the growth and development of hair follicles and fiber traits in sheep.

## 4. Discussion

A large number of studies have shown that lncRNAs play an important role in cancer [28,29]; however, there are few studies on their potential role in the growth and development of mammalian hair follicles and traits. In order to explore the potential effects of lncRNAs on sheep hair traits among different breeds, in this study, skin tissues from SM and STH sheep were observed via histomorphology. There are significant differences in hair follicle characteristics between the two sheep breeds studied, suggesting that there may be a close relationship between hair follicle performance and wool traits. Through high-throughput sequencing, we further identified DE lncRNAs and mRNAs and combined the results with multiple structural characteristics to understand the data. As is consistent with previous studies, the findings indicated that the lncRNA expression level was lower and less evolutionarily conserved than that of mRNA. lncRNAs often contained fewer exons than protein-coding transcripts, and because of this, the lengths were shorter than those of mRNAs; in addition, the ORF of lncRNAs was shorter than that of mRNAs [8,30]. As confirmed through qRT-PCR, the trend in DE gene expression aligned with the findings from RNA-seq. In the results of GO and KEGG enrichment analyses, the target genes of DE lncRNAs were significantly enriched in epidermis and skin development, PI3K-Akt, Ras, MAPK, and other GO terms and signaling pathways. At the same time, this overlapped with the enrichment results of DE mRNAs. These results suggest that DE lncRNAs and target genes may play important roles in regulating hair follicle growth and development.

Research indicates that the primary components of hair follicle fibers are keratin (*KRT*) and keratin-associated protein (*KRTAP*) [30,31,32]. Sun H et al. found that *KRTAP6-1* and *KRTAP6-5* may be involved in regulating the fiber diameter and staple strength of Gansu Alpine fine-wool sheep [33]. Moreover, most differentially expressed *KRT* and *KRTAP* genes (*KRT38*, *KRTAP15-1*, and *KRTAP3-1*) are involved in hair fiber regulation, and their expression is higher in the growth stage than in the middle development stage [9]. Zhang et al. showed that *KRT85*, *KRTAP15-1*, and *KRTAP3-1* may be key to fiber diameter differences [34]. In this study, the lncRNA *MSTRG.9225.1* and its cis-target gene *KRTAP15-1* and trans-target gene *KRT10* were also found to be significantly higher in STH than in SM. Meanwhile, the cis-target gene *KRTAP15-1* of the lncRNA *MSTRG.9225.1* was significantly enriched during keratinization and epidermal cell differentiation as well as epithelium and skin development; the trans-target gene *KRT10* was significantly enriched in keratin differentiation, and the above GO terms were closely related to the growth and development of HF and fiber characteristics [4,35]. Therefore, it is speculated that the lncRNA *MSTRG.9225.1* interacts with the cis-target gene *KRTAP15-1* or the trans-target gene *KRT10* to participate in the regulation of hair follicle growth and development and fiber traits.

The *FGF* gene family, which is extensively present in skin tissues, is crucial for the proliferation, differentiation, and periodic growth of DPCs [36]. Moreover, *FGF1*, *FGF7*, and *FGF22* in hair follicle cells have been shown to regulate hair growth and development [37,38]. In their study, Kwack et al. indicated that numerous growth factors and intercellular signaling molecules in HFs, including *IGF1*, *KGF*, *HGF*, and others, are produced and released by DPCs, playing a role in adjacent cells to regulate proliferation, differentiation, and the extension of hair shafts [39]. Some studies have also shown that *IGF* exists in the inner root sheath and medulla. It affects hair follicle cell proliferation and the hair growth cycle [40]. In this study, the expression levels of the lncRNAs *MSTRG.98769.1* and *MSTRG.84658.1*, and the target genes *FGF1* and *IGF1* in STH skin tissues, were also significantly higher than those of SM. In the enrichment analysis of trans-target genes, *FGF1* and *IGF1* were significantly enriched in the Ras and MAPK signaling pathways, which are known to be related to hair follicle growth and development [41,42]. The MAPK signaling pathway regulates skin and hair follicle development, epidermal and keratinocyte differentiation, wool cycle growth, and wool fiber quality [43,44]. Meanwhile, in the enrichment analysis of differentially expressed mRNAs, *FGF1* and *IGF1* were found to be significantly enriched in the PI3K-Akt signaling pathway, which plays a vital role in sustaining and reinstating hair growth in dermal papilla cells, as well as in encouraging their multiplication and preventing their programmed cell death [6]. Chen et al. found that inhibition of PI3K or Akt with specific inhibitors could significantly inhibit cultured epidermal stem cells and skin-derived precursor-mediated hair follicle regeneration [45]. Yamane et al. found that inhibition of the PI3K/Akt signaling pathway decreased the hair-inducing ability of DPCs [46]. These findings suggest that the PI3K/Akt signaling pathway may play a role in promoting the growth and development of hair follicles. The growth and development of hair follicles are influenced by dermal papilla cells, and these differentially expressed target genes may play an important role in the proliferation or apoptosis of dermal papilla cells. Therefore, it is speculated that lncRNA *MSTRG.98769.1* and *MSTRG.84658.1* indirectly participate in the proliferation or apoptosis of dermal papilla cells by regulating the expression of their respective target genes, thus affecting the growth and development of hair follicles.

**Figure 14:**
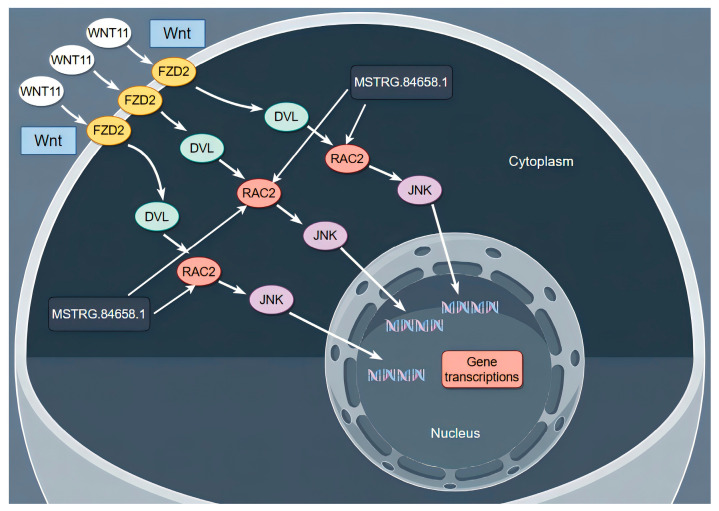
Schematic representation of the effects of *MSTRG.84658.1* and *RAC2* on the expression of related proteins in the Wnt signaling pathway.

At present, there is no relevant study on the effect of *RAC2* on sheep dermal papilla cells. In this study, the KEGG enrichment results showed that *RAC2* is enriched in the Wnt signaling pathways. Wnt signaling is thought to be central to hair follicle morphogenesis and regeneration [47,48]. Zhu et al. found that biglycan plays a key role in regulating HF regeneration in paracrine and autocrine ways by activating the Wnt signaling pathway [49]. These studies suggest that the Wnt signaling pathway plays an important role in the growth and development of hair follicles. Therefore, as shown in Figure 14, we speculate that *MSTRG.84658.1* participates in the Wnt signaling pathway by targeting *RAC2*, indirectly regulating the molecular mechanism of dermal papilla cell function.

## 5. Conclusions

In this study, a transcriptome comparison of the skin tissues of Super Merino and Small-Tailed Han sheep was conducted to further identify a large number of DE lncRNAs and DE mRNAs that may affect hair follicle growth, development, and fiber traits. *MSTRG.9225.1* and *MSTRG.98769.1* may interact with their respective target genes, *KRTAP15-1*, *FGF1*, and *IGF1*, and these interactions may be involved in regulating hair follicle growth, development, and fiber traits. In addition, by targeting *RAC2*, *MSTRG.84658.1* may regulate the Wnt signaling pathway involved in the development of sheep hair follicles. This study provides a theoretical reference for the improvement of sheep breeding in the future, laying a foundation for further research on the effects of *MSTRG.84658.1* and the target gene *RAC2* in DP cells.

## Figures and Tables

**Table 1:** Primer sequences used in qRT-PCR.

Gene	Primer Sequence 5′→3′	Product Length (bp)
*MSTRG.93521.1* (down)	CAGGCTCGGCTGAGGTTTGG AGTCTGTGGTTGTGTCCAGTGATG	114
*MSTRG.20995.1* (down)	GTGGGGAGCCAAAGAGGAAT AGGTCAACGGCGTGGTTAAA	165
*MSTRG.154129.25* (up)	TGGTTAGCTCAAGGTTCGAGA GCAGCAGGAGGAAACAACCTA	141
*MSTRG.96951.1* (down)	ACTCGCCTTGTTCCAACCTC AGTGTGTCCGAATCTGCCTC	131
*MSTRG.31806.12* (up)	AGCAAGAGCCAGTGGTACAATA ATAGCCTCCAGCAGACGAGG	92
*COL7A1* (up)	ATGATCCCTGTTCGCTTCCAC TCCGCAGCCACCATAGACGA	122
*CNN1* (down)	AGAACACCAACCACACGCAAG GCCCGATAATGTTCCGCCCTT	159
*KLK6* (up)	AGAGACTGCTCAGCCAACCAC TGCTTCTCATCCCCGGCACAC	179
*IGFBP5* (down)	CGCCACTCATTTCATCTCATGT TGCGACCTTGCCAGAGATTC	94
*CRABP1* (down)	ACGGGGACCAGTTCTACATCA TCCCAAGTGGGTAAGCTCCTG	127
*KRT10* (down)	AGCAGAAACTAGCTGGGATACT AGGACTCTACCATCAGGTGC	81
*GAPDH*	GTGGACCTGACCTGCCGTCTAG GAGTGGGTGTCGCTGTTGAAGTC	149

**Table 2:** Comparison of the differences in skin structure and hair follicle characteristics (mean ± SD) of the Super Merino and Small-Tailed Han sheep.

Traits	Super Merino (*n* = 6)	Small-Tailed Han Sheep (*n* = 6)
Skin thickness (µm)	1355.37 ± 50.48	1616.57 ± 44.53 **
Hair follicle density (mm^2^)	19.74 ± 2.04 **	11.59 ± 1.13
Diameter of primary dermal papilla (µm)	122.35 ± 2.99	143.68 ± 5.46 **
Diameter of secondary dermal papilla (µm)	49.26 ± 1.99	63.81 ± 3.24 **
S/P	19.65 ± 2.90 **	5.46 ± 2.43

“*n*” represents the number of samples; “**” = *p*-value < 0.01.

**Table 3:** Quality statistics of the sequencing data.

BMK-ID	Total Reads	Mapped Reads	Uniquely Mapped Reads	Multiple Mapped Reads	Q20 (%)	Q30 (%)
SM1	119,612,582	113,676,411 (95.04%)	111,058,913 (92.85%)	2,617,498 (2.19%)	97.67	93.62
SM2	115,429,242	109,531,249 (94.89%)	107,228,260 (92.90%)	2,302,989 (2.00%)	97.76	93.79
SM3	124,719,310	117,304,477 (94.05%)	115,270,935 (92.42%)	2,033,542 (1.63%)	97.86	93.87
SM4	118,615,420	113,129,111 (95.37%)	109,835,326 (92.60%)	3,293,785 (2.78%)	97.67	93.62
SM5	132,926,276	127,869,012 (96.20%)	125,542,321 (94.45%)	2,326,691 (1.75%)	97.85	93.87
SM6	122,335,752	117,949,087 (96.41%)	115,542,600 (94.45%)	2,406,487 (1.97%)	98.16	94.68
STH1	127,399,620	123,042,683 (96.58%)	120,295,753 (94.42%)	2,746,930 (2.16%)	98.08	94.52
STH2	127,087,066	122,677,440 (96.53%)	119,419,027 (93.97%)	3,258,413 (2.56%)	98.18	94.80
STH3	128,176,154	123,199,306 (96.12%)	119,827,700 (93.49%)	3,371,606 (2.63%)	98.07	94.45
STH4	132,012,936	126,513,200 (95.83%)	123,006,721 (93.18%)	3,506,479 (2.66%)	97.84	93.88
STH5	117,224,636	112,912,295 (96.32%)	110,197,340 (94.01%)	2,714,955 (2.32%)	97.89	93.96
STH6	114,176,126	109,862,331 (96.22%)	107,334,555 (94.01%)	2,527,776 (2.21%)	98.02	94.38

ID: sample number. Total Reads: number of clean reads in a single end. Mapped Reads: number of reads mapped to the reference genome and percentage of clean reads. Uniq Mapped Reads: number of reads aligned to unique locations in the reference genome and percentage of clean reads. Multiple Mapped Reads: the number and percentage of clean reads mapped to multiple locations in the reference genome. Q20 (%): clean data and the percentage of bases with a mass value greater than or equal to Q20. Q30 (%): clean data and the percentage of bases with a mass value greater than or equal to Q30.3.1. Subsection.

**Table 4:** Comparison of expression trends of RNA-seq and qRT-PCR.

ID	RNA-seq (Regulated)	qRT-PCR (Regulated)
*MSTRG.96951.1*	down	down
*MSTRG.31806.12*	up	up
*MSTRG.20995.1*	down	down
*MSTRG.93521.1*	down	down
*MSTRG.154129.25*	up	up
*CRABP1*	down	down
*KRT10*	down	down
*CNN1*	down	down
*COL7A1*	up	up
*KLK6*	up	up
*IGFBP5*	down	down

ID: Gene name.RNA-seq(regulated): Gene expression trends in RNA-seq results. qRT-PCR(regulated): Gene expression trends in qRT-PCR results. “up” indicates the gene high expression in the skin tissue of SM. “down” indicates the gene high expression in the skin tissue of STH.

## Data Availability

The data presented in this study are available upon request from the corresponding author.
